# Molybdenum Supply Alleviates the Cadmium Toxicity in Fragrant Rice by Modulating Oxidative Stress and Antioxidant Gene Expression

**DOI:** 10.3390/biom10111582

**Published:** 2020-11-21

**Authors:** Muhammad Imran, Saddam Hussain, Mohamed A. El-Esawi, Muhammad Shoaib Rana, Muhammad Hamzah Saleem, Muhammad Riaz, Umair Ashraf, Mouloumdema Pouwedeou Potcho, Meiyang Duan, Imran Ali Rajput, Xiangru Tang

**Affiliations:** 1 State Key Laboratory for Conservation and Utilization of Subtropical Agro-Bioresources, College of Agriculture, South China Agricultural University, Guangzhou 510642, China; muhammadimran@scau.edu.cn (M.I.); gilbert@stu.scau.edu.cn (M.P.P.); meiyang@scau.edu.cn (M.D.); 2 Scientific Observing and Experimental Station of Crop Cultivation in South China, Ministry of Agriculture and Rural Affairs, Guangzhou 510642, China; 3 Guangzhou Key Laboratory for Science and Technology of Fragrant Rice, Guangzhou 510642, China; 4 Department of Agronomy, University of Agriculture, Faisalabad 38040, Punjab, Pakistan; shussain@uaf.edu.pk; 5 Botany Department, Faculty of Science, Tanta University, Tanta 31527, Egypt; mohamed.elesawi@science.tanta.edu.eg; 6 Key Laboratory of Arable Land Conservation (Middle and Lower Reaches of Yangtze River), Ministry of Agriculture and Rural Affairs, Huazhong Agricultural University, Wuhan 430070, China; muhammadshoaib@webmail.hzau.edu.cn; 7 College of Plant Science and Technology, Huazhong Agricultural University, Wuhan 430070, China; saleemhamza312@webmail.hzau.edu.cn; 8 Root Biology Center, College of Natural Resources and Environment, South China Agricultural University, Guangzhou 510642, China; riaz1480@scau.edu.cn; 9 Department of Botany, Division of Science and Technology, University of Education, Lahore 54770, Punjab, Pakistan; umair.ashraf@ue.edu.pk; 10 Pakistan Agricultural Research Council (PARC) Arid Zone Research Institute, Umerkot 69100, Sindh, Pakistan; ranaimran234@gmail.com

**Keywords:** molybdenum, cadmium toxicity, cadmium alleviation, oxidative stress, ROS, gene expression, fragrant rice

## Abstract

Increasing evidence shows that cadmium (Cd) toxicity causes severe perturbations on growth performance, physio-biochemical and molecular processes in crop plants. Molybdenum (Mo), an essential trace element, plays key roles in oxidative stress tolerance of higher plants. Hence, the present study has been conducted to investigate the possible role of Mo in alleviating Cd-induced inhibitions in two fragrant rice cultivars namely Guixiangzhan (GXZ) and Meixiangzhan-2 (MXZ-2). The results revealed that Mo application enhanced the plant dry biomass by 73.24% in GXZ and 58.09% in MXZ-2 under Cd stress conditions, suggesting that Mo supplementation alleviated Cd-induced toxicity effects in fragrant rice. The enhanced Cd-tolerance in fragrant rice plants prompted by Mo application could be ascribed to its ability to regulate Cd uptake and reduce Cd-induced oxidative stress as evident by lower hydrogen peroxide levels, electrolyte leakage and malondialdehyde contents in Cd-stressed plants. The ameliorative role of Mo against Cd-toxicity also reflected through its protection to the photosynthetic pigments, proline and soluble protein. Mo also induced antioxidant defense systems via maintaining higher contents of glutathione and ascorbate as well as enhancing the ROS-detoxifying enzymes such as catalase, peroxidase, superoxide dismutase and ascorbate peroxidase activities and up-regulating transcript abundance in both fragrant rice cultivars under Cd stress. Conclusively, Mo-mediated modulation of Cd toxicity in fragrant rice was through restricting Cd uptake, maintaining photosynthetic performance and alleviating oxidative damages via the strong anti-oxidative defense systems; however, GXZ cultivar is comparatively more Cd tolerant and Mo-efficient as evident from the less growth inhibition and biomass reduction as well as enhanced Mo-induced Cd stress tolerance and less oxidative damage than MXZ-2 fragrant rice cultivar.

## 1. Introduction

Agricultural sustainability, food production and crop productivity are not safe due to the persistent accumulation of heavy metals (non-essential plant elements) in soil profiles. Such polluted soils with various heavy metal pools have decreased plant growth by affecting the different physiological, biochemical and molecular pathways [1,2]. Nevertheless, the degree of growth reduction depends on experimental conditions, heavy metals and plant species [3,4]. Previous studies on various heavy metals; cadmium (Cd), nickel (Ni), lead (Pb), chromium (Cr) and zinc (Zn) reported that the highest toxic reaction among different heavy metals was observed in the presence of Cd in wheat [2], blackgram [5] and rice [6].

Cadmium toxicity has become a serious threat in agricultural soils around the world [7]. Due to its non-essential form in living organisms, the consequences of Cd toxicity are very cumbersome for both animals and plants even at low concentrations. In paddy soils, Cd is taken up by plant roots and then transferred to above ground parts and affects plants morphologically, physiologically and biochemically, from germination to subsequent growth and developmental stages [8]. The most common morphological symptoms of Cd infected plants include stunted root and shoot growth, extreme reduction in normal accumulation of biomass, leaf chlorosis and eventually plant death [1,8]. Previous studies have revealed that absorption, aggregation and transfer of micro- and macro-nutrients to various plant parts have been significantly affected by Cd toxicity [9,10,11]. These interferences between Cd and other essential macro/micronutrients could be due to some kind of molecular rivalry in nutrient uptake channels or in plant metal transporters [10].

Molybdenum (Mo), a necessary trace element for higher plants, plays a vital role in various plant physio-biochemical processes such as root growth, water utilization, photosynthesis, chlorophyll biosynthesis, chloroplast configuration and ultra-structural integrity, efficient N assimilation and utilization, and biosynthesis of endogenous hormones [12,13,14,15]. On the other hand, Mo has also been widely reported as a stress-resistant element to promote the reinforcement of oxidative stress tolerance to salinity [16], drought [17], low temperature [14] and ammonium stresses [18]. However, plants are equipped with various pathways to stabilizing ROS homeostasis by enhanced activities and transcribing the abundance of enzymatic and non-enzymatic antioxidants. The dynamic antioxidant defense system, therefore, works in concert to alleviate oxidative lesions in plant cells and detoxify undue ROS output in stressful environments [19,20,21]. Similarly, some plant studies have also recently documented the beneficial effects of Mo application to relieve Cd stress injuries in *Ricinus communis* L. [22] and *Brassica napus* L. [11]. Nevertheless, Mo-induced effects on growth and physio-biochemical responses of fragrant rice cultivars under Cd toxicity have not yet been recorded in any of the studies.

Fragrant rice is the best quality rice type, and is known worldwide for its distinctive aroma and flavor [23]. Our previous experiments have reported the detrimental effects of Cd stress on rice yield and other related components, i.e., number of panicles, spikelet per panicle, seed setting rate, weight of 1000-grains, quality of aroma (rice grain fragrance) and yield of grains [24,25]. Nevertheless, here we will research how Mo (an essential and anti-stress trace element) supplementation could modulate Cd toxicity effects on physio-biochemical processes in fragrant rice at the seedling stage.

Therefore, the aims of the current experiment were to study the Mo-induced effects on growth and physio-biochemical attributes in two Cd-stressed fragrant rice cultivars and identify (1) whether Mo supplementation alleviates Cd-induced growth inhibition and oxidative stress, and (2) these effects could be connected to major antioxidant enzyme activities and related gene expression. To explore this hypothesis, a combination of physio-biochemical and molecular approaches was used to assess Mo-induced Cd stress tolerance by evaluating plant growth and biomass, soluble protein, proline and photosynthetic pigment contents, Cd uptake, hydrogen peroxide (H_2_O_2_), malondialdehyde (MDA), and electrolyte leakage (EL), as well as levels/activities of antioxidants and representative encoding genes in two fragrant rice cultivars.

## 2. Materials and Methods

### 2.1. Plant Husbandry and Growth Conditions

Two fragrant rice cultivars (*Oryza sativa* L.) Guixiangzhan (GXZ) and Meixiangzhan-2 (MXZ-2) were obtained from South China Agricultural University, Guangzhou-China. These fragrant rice cultivars were selected based on their similar growth period but differential responses to Cd toxicity in field trials conducted in 2015 and 2016 [24,25]. GXZ cultivar was identified as more Cd tolerant than MXZ-2 rice cultivar due to less Cd uptake and distribution to different plant parts under the same conditions. Both the fragrant rice cultivars seeds were first surface sterilized, to minimize contamination, with 2.5% NaOCl solution and rinsed three times with distilled water and soaked for 24 h. Prior to use, all cultural instruments used during this hydroponic research were sterilized (autoclaved). After that, the seeds were placed on the cheese cloth for germination in a growth chamber (28 ± 2 °C) for 7 days and uniform sized rice seedlings were transferred to plastic pots containing one-quarter strength Hoagland solution in a controlled environmental growth chamber with temperature; 28 ± 2 °C, relative humidity (R.H.); 65–70% and photon density; 820 mmol m^−2^ s^−1^. Rice seedlings were fixed into the perforated lids of the containers with small sponges and grown in 1/4 and 1/2 strength Hoagland solution for the first and second 2-days interval, respectively. Subsequently, full strength Hoagland solution was applied along with Mo and Cd treatments for the next 10 days until 20 days-old rice seedlings were harvested. As defined by the International Rice Research Institute [26], the Hoagland Nutrient Solution contained 1 mM (NH_4_)_2_SO_4_, 1 mM KH_2_PO_4_, 1 mM Ca(NO_3_)_2_·4H_2_O, 1 mM MgSO_4_·7H_2_O, 2 mM Na_2_SiO_3_·9H_2_O, 20 µM Fe-EDTA, 1 µM ZnSO_4_·7H_2_O, 9.1 µM MnSO_4_, 0.1 µM CuSO_4_·5H_2_O and 10 µM H_3_BO_3_. Combinations of treatments included two levels of Mo (0 and 1 µM [(NH_4_)_6_Mo_7_O_24_·4H_2_O]) and two levels of Cd (0 and 100 µM CdCl_2_) applied to two fragrant rice cultivars at pH 6.0 ± 0.05. During the course of the study the Hoagland nutrient solutions were refreshed after every two days. The experiment was conducted under completely randomized design (CRD) arrangement with four independent replicates.

### 2.2. Morphological Traits

The growth parameters of both rice seedlings were estimated in terms of plant fresh and dry biomass, roots and shoot lengths. Five seedlings from each treatment were randomly selected and dissected into roots and shoot. The root and shoot lengths were measured by meter scale. Fresh weight was immediately recorded following harvest. Then seedlings were first dried for 0.5 h at 90 °C and then for 72 h at 65 °C and weighed using digital equilibrium for dry biomass measurement.

### 2.3. Measurement of Photosynthetic Pigments

The leaves of both fragrant rice cultivars were used to calculate chlorophyll a, b and carotenoids contents by following our previously described protocol [15]. Briefly, rice leaves were extracted by ethanol (95%) and absorption was recorded at 649, 665 and 470 nm using a spectrophotometer (UV-VIS 2550, Shimadzu, Japan).

### 2.4. Determination of Cadmium and Molybdenum Concentrations

The oven dried plant samples were ground and digested in 5:1 (*v*/*v*) HNO_3_:HClO_4_ (5 mL) in a microwave oven (MLS 1200, Milestone, FKV, Boldone, Italy). The Cd and Mo concentrations were measured in the digested samples by inductively coupled plasma (ICP)-optical emission spectroscopy (Vista-PRO, Varian, Inc., Palo Alto, California, CA, USA) and ICP-mass spectrometry (ICP-MS) (ELAN DRC-e, Perkin-Elmer Sciex, Wilmington, DE, USA) [27,28].

### 2.5. Soluble Protein and Proline Determination

The Bradford method (1976) was adopted for estimating the soluble protein (S-protein) content [29] using bovine serum albumin (BSA). Proline levels were calculated by using the previously described protocol of [30].

### 2.6. Measurement of H_2_O_2_, MDA and Electrolyte Leakage

The previously described method of [17] was used to quantify hydrogen peroxide (H_2_O_2_) contents in rice leaves. Briefly, fresh rice leaf samples were ground with trichloroacetic acid (0.1% *w*/*v*) and centrifuged (20 min, 12,000 rpm). The reaction mixture contained supernatant, potassium phosphate buffer (100 mM, pH 6.8) and potassium iodide (1M). The reaction mixture was incubated (1 h) in darkness and absorbance was recorded at 390 nm. Malondialdehyde (MDA) contents were measured by following our previously describe protocol [18]. Electrolyte leakage (EL) was measured by keeping fresh leaf samples in water containing closed vials (6 h, 25 °C) and reading was noted as EC1 on an EC meter (SX-650, Sansin, China). To record EC2, samples were again incubated (2 h) at 90 °C and cooled to 25 °C for reading on the EC meter. The EL in leaf tissues was calculated as: EL (%) = (EC1/EC2 × 100) [31].

### 2.7. Measurement of Enzymatic and Non-Enzymatic Antioxidants

Fresh leaf samples were homogenized with sodium phosphate buffer (50 mM, pH 7.8) and centrifuged for 10 min at 12,000 rpm (4 °C). The supernatant was separated from crude fibers for the determination of catalase (CAT; EC 1.11.1.6), superoxide dismutase (SOD; EC 1.15.1.1), ascorbate peroxidase (APX; EC 1.11.1.11) and peroxidase (POD; EC 1.11.1.7) activities by following our previously described methods [18]. For the determination of ascorbate (AsA) contents in the rice leaves, fresh samples were normalized with TCA (10 % *w*/*v*) and centrifuged at 15,000 rpm for 15 min (4 °C). The supernatant was separated for estimating AsA concentration following the previously described protocol [32]. Reduced glutathione (GSH) and oxidized glutathione (GSSG) contents in leaf tissues were measured by using ‘A006-1’ and ‘A061-2’ kits following the instructed manuals, respectively, purchased from Nanjing Jiangcheng Bioengineering Institute (www.njjcbio.com) China. 

### 2.8. Total RNA Extraction and qRT-PCR Analysis

Frozen rice leaf tissues were used for total RNA extraction and subsequently qRT-PCR analysis by following our previously described procedure [33]. Briefly, TRIzol reagent (Invitrogen, Carlsbad, CA, USA) was used for RNA extraction and then dissolved in DEPC•H_2_O and quantity was measured with spectrophotometer Nano Drop UV-VIS 2000 (Thermo Fisher Scientific, Waltham, MA, USA). M-MLVRTase (Promega, Madison, WI, USA), Oligo (dT18) primers (Promega, Madison, WI, USA) and dNTP were mixed with quantified RNA to produce cDNA through IQ5 Real-Time PCR (Bio-Rad, California, CA, USA). For subsequent detections, gene-specific primers, synthesized cDNA templates and SYBR Green mix (Bio-Rad, Hercules, CA, USA) were mixed together into a 96-well plate and subjected to succeeding program: 95 °C for 30 s to denature DNA, 40 cycles for 20 s (95 °C) and 20 s at annealing temperatures (Tm) of respective primers (Table 1), followed by 72 °C for 30 s. For relative quantification (RQ), rice *ACTIN* (*Os03g50885*) (F) 5′-TGCCAAGGCTGAGTACGACGA-3′ and (R) 5′-CAAGCAGGAGGACGGCGATA-3′ was used as the housekeeping gene [19]. The full information of gene index number, primers sequences and annealing temperatures of genes of interest is available in (Table 1). Three biological replicates were used and the expression levels were measured by standardizing the Ct value for each gene relative to the Ct value of ACTIN, and the 2-ΔΔCt method was used for quantification [18,34].

### 2.9. Statistical Analysis

The data for both rice cultivars were tested by employing three-way ANOVA using statistical package Statistix 8.1 (Analytical software, Tallahassee, Florida, FL, USA). Mean variances were separated by LSD-test (*p* < 0.05). Sigmaplot 10.0 was used for graphical representations.

## 3. Results

### 3.1. Effects of Mo Application on Plant Growth and Photosynthetic Pigments Contents under Cd Toxicity

Results indicated that Mo deprived (–Mo) rice seedlings with or without Cd treatments had smaller root and shoot lengths, fresh and dry weights as compared to those grown in sufficient +Mo treatments (Table 2).

**Figure 1:**
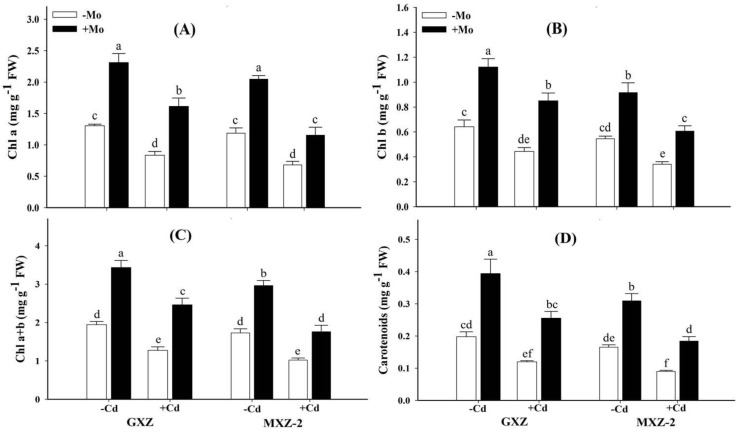
Influence of molybdenum (Mo) and cadmium (Cd) on; (**A**) chlorophyll a (Chl a); (**B**) chlorophyll b (Chl b); (**C**) total chlorophyll (Chl a+b) and (**D**) carotenoids contents in leaves of two fragrant rice Guixiangzhan (GXZ) and Meixiangzhan-2 (MXZ-2) cultivars. Both fragrant rice cultivars were treated with two molybdenum levels: 0 µM (–Mo) and 1 µM (+Mo), against two cadmium levels: 0 µM (−Cd) and 100 µM (+Cd) in modified Hoagland solution. Vertical bar above indicates standard error of four replicates. Different lowercase letters (a, b, c, etc.) represent significant differences according to the LSD-test (*p* < 0.05, *n* = 4).

The minimum growth of both rice cultivars was observed under Cd stress in Mo deprived seedlings; however, the negative effects of Cd toxicity and Mo deprivation were more severe in MXZ-2 relative to GXZ rice cultivar, indicating that GXZ is comparatively more tolerant to Cd stress than MXZ-2 (Table 2). Under Cd treatment, sufficient Mo supply (Mo+) increased the plant dry weight by 73.24% and 58.09% in GXZ and MXZ-2, respectively, compared with –Mo treatment, suggesting that GXZ showed higher performance and is more responsive to Mo supply than MXZ-2 fragrant rice cultivar. Furthermore, in the absence of Mo, the photosynthetic pigment contents (Chl a, Chl b, Chl a+b and carotenoids) were significantly decreased under Cd toxicity in both rice cultivars (Figure 1), while the combined application of Mo and Cd significantly enhanced their contents under Mo + Cd+ treated rice plants, suggesting that Mo supplementation alleviated Cd toxicity. Compared with Mo − Cd+ treatment, contents of Chl a+b and carotenoids were improved by 92.79% and 113.02% in GXZ while 72.18% and 104.78% in MXZ-2 rice seedlings, respectively, under combined Mo + Cd+ treatment (Figure 1), indicating that Cd toxicity caused more severe disruption in photosynthetic pigments in MXZ-2 relative to GXZ cultivar and Mo supply alleviated Cd-induced inhibitory effects and resumed photosynthetic pigments in fragrant rice.

### 3.2. Molybdenum and Cadmium Concentrations in Plant Parts

The Mo concentrations in roots and shoot tissues were significantly enhanced with Mo application while non-significant effects were recorded in Mo concentration under with or without Cd toxicity in both rice cultivars. Moreover, GXZ accumulated more Mo concentration in plant tissues than MXZ-2 cultivar (Table 3), suggesting that GXZ might be more efficient in Mo uptake, translocate and utilization than MXZ-2 fragrant rice cultivar. Cd concentrations were increased in roots and shoots under Cd toxicity with or without Mo treatments; however, Cd concentrations were higher in roots than shoots in both fragrant rice cultivars. Moreover, compared with Mo deprived (Mo − Cd+) treatment, Cd concentrations were decreased by 24.66% and 17.99% in shoots while 20.92% and 15.11% in roots of GXZ and MXZ-2 rice cultivars, respectively, under combined (Mo + Cd+) treatment (Table 3), indicating that Mo supplementation reduced Cd uptake in fragrant rice. 

### 3.3. Effect of Mo on the S-Protein and Proline Contents under Cd Toxicity

**Figure 2:**
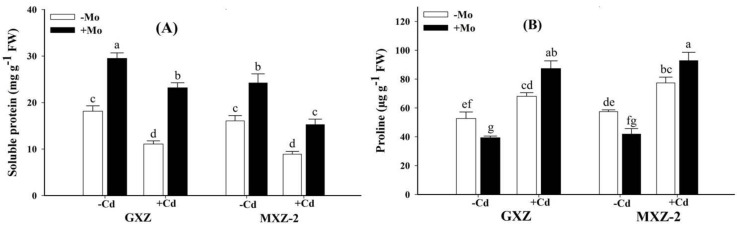
Influence of molybdenum (Mo) and cadmium (Cd) on (**A**) soluble protein, and (**B**) proline contents in leaves of two fragrant rice Guixiangzhan (GXZ) and Meixiangzhan-2 (MXZ-2) cultivars. Both fragrant rice cultivars were treated with two molybdenum levels: 0 µM (–Mo) and 1 µM (+Mo), against two cadmium levels: 0 µM (−Cd) and 100 µM (+Cd) in modified Hoagland solution. Vertical bar above indicates standard error of four replicates. Different lowercase letters (a, b, c, etc.) represent significant differences according to the LSD-test (*p* < 0.05, *n* = 4).

S-protein and proline contents were significantly changed under combined application of Mo and Cd in leaf tissues of both fragrant rice seedlings (Figure 2). The Mo deprivation recorded significantly lower S-protein content with or without Cd stress relative to Mo+ treatment; however, the lowest content of S-protein was measured for combined Mo − Cd+ treatment in both fragrant rice cultivars (Figure 2A). Under Cd stress, Mo application increased the S-protein content by 109.18% and 71.35% in GXZ and MXZ-2 rice cultivars, compared with –Mo treatment (Figure 2A), indicating that Mo supply significantly prevented the S-protein reduction under Cd-stressed conditions. In contrast to S-protein, proline contents were significantly increased with or without Cd stress as compared with Mo + Cd− treated rice seedlings. Compared with sufficient Mo supply (Mo+), Cd toxicity (Mo − Cd+) increased proline contents by 73.03% and 84.98% in GXZ and MXZ-2 rice cultivars, respectively (Figure 2B). Interestingly, concurrent supply (Mo + Cd+) further accumulated proline by 28.28% and 19.91% in GXZ and MXZ-2 rice cultivars, respectively, over Cd stressed (Mo − Cd+) treatment (Figure 2B).

### 3.4. Influence of Molybdenum and Cadmium on the Contents of H_2_O_2_, MDA and EL in Rice Plants

**Figure 3:**
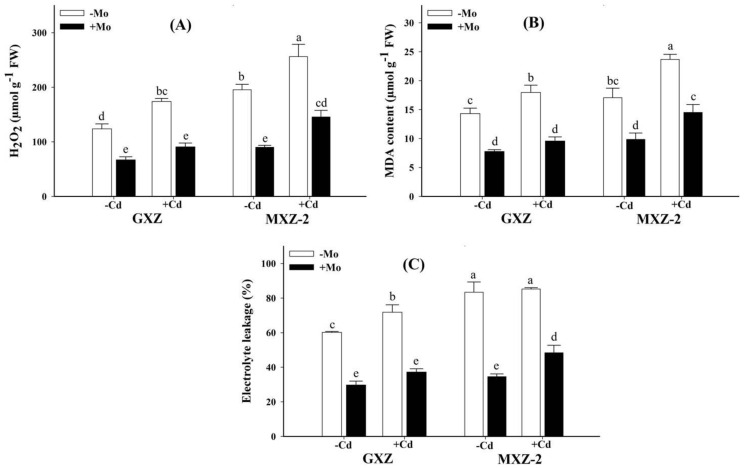
Influence of molybdenum (Mo) and cadmium (Cd) on; (**A**) hydrogen peroxide (H_2_O_2_); (**B**) malondialdehyde (MDA) and (**C**) electrolyte leakage (EL) in leaves of two fragrant rice Guixiangzhan (GXZ) and Meixiangzhan-2 (MXZ-2) cultivars. Both fragrant rice cultivars were treated with two molybdenum levels: 0 µM (–Mo) and 1 µM (+Mo), against two cadmium levels: 0 µM (−Cd) and 100 µM (+Cd) in modified Hoagland solution. Vertical bar above indicates standard error of four replicates. Different lowercase letters (a, b, c, etc.) represent significant differences according to the LSD-test (*p* < 0.05, *n* = 4).

Results showed that Mo supply (Mo+) significantly reduced contents of H_2_O_2_, MDA and EL with or without Cd toxicity in both rice cultivars while Cd toxicity significantly enhanced H_2_O_2_, MDA and EL in MXZ-2 than GXZ rice cultivar (Figure 3), indicating that MXZ-2 rice cultivar is comparatively more susceptible to Cd stress than GXZ. Compared with sufficient Mo+ treatment, Cd stress significantly increased contents of H_2_O_2_ by 159.94% and 184.79%, MDA by 131.00% and 140.60% and EL by 141.66% and 146.74%, respectively, in GXZ and MXZ-2 rice seedlings (Figure 3). However, compared with Mo + Cd− treatment, lower rise under combined (Mo + Cd+) supply in H_2_O_2_ contents (35.89% and 61.92%), MDA contents (23.01% and 47.45%) and EL (25.33% and 39.97%), respectively, in GXZ and MXZ-2 fragrant rice seedlings indicates that Mo has a significant role in ameliorating Cd toxicity in fragrant rice.

### 3.5. Effects of Mo Supplementation on Enzymatic and Non-Enzymatic Antioxidant Activities in Cd-Stressed Rice Seedlings

**Figure 4:**
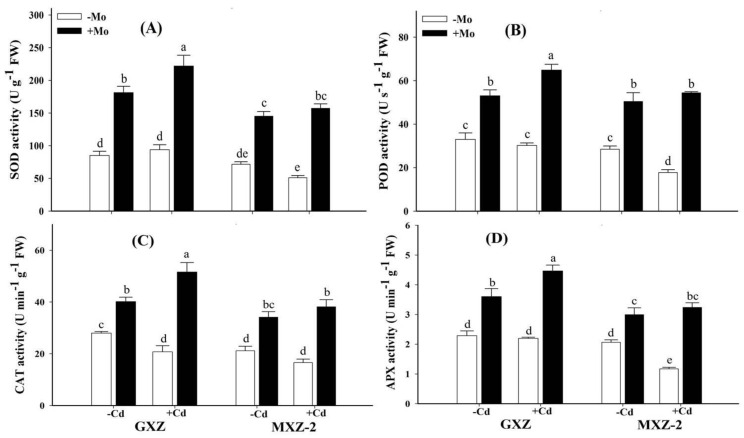
Influence of molybdenum (Mo) and cadmium (Cd) on enzymatic antioxidants; (**A**) superoxide dismutase (SOD), (**B**) peroxidase (POD), (**C**) catalase (CAT), and (**D**) ascorbate peroxidase (APX) activities in leaves of two fragrant rice Guixiangzhan (GXZ) and Meixiangzhan-2 (MXZ-2) cultivars. Both fragrant rice cultivars were treated with two molybdenum levels: 0 µM (–Mo) and 1 µM (+Mo), against two cadmium levels: 0 µM (−Cd) and 100 µM (+Cd) in modified Hoagland solution. Vertical bar above indicates standard error of four replicates. Different lowercase letters (a, b, c, etc.) represent significant differences according to the LSD-test (*p* < 0.05, *n* = 4).

To investigate the potential role of Mo supplementation to counteract Cd-induced oxidative stress in fragrant rice plants, various antioxidant enzyme activities were determined (Figure 4). The results demonstrated that under Mo deprivation, Cd stress significantly inhibited the antioxidant enzymes activities as compared to Mo + Cd− treatment in both rice cultivars; however, a more severe decrease was observed in MXZ-2 indicating that it might be comparatively more sensitive to Cd toxicity (Figure 4).

**Figure 5:**
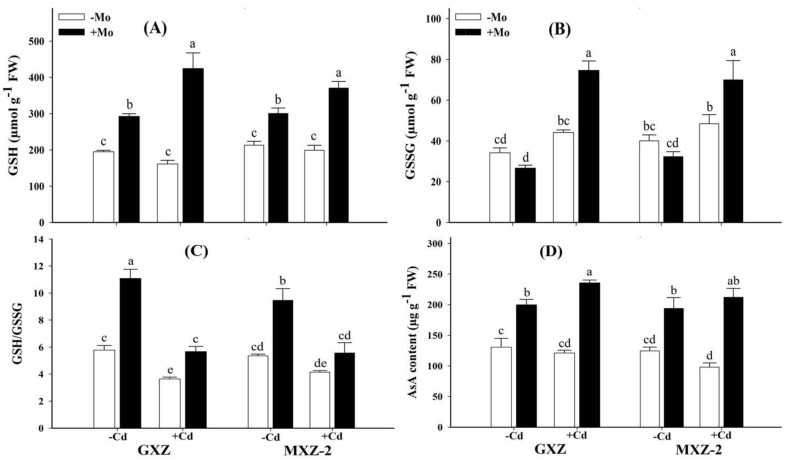
Influence of molybdenum (Mo) and cadmium (Cd) on; (**A**) reduced glutathione (GSH), (**B**) oxidized glutathione (GSSG), (**C**) ratio of GSH/GSSG and (**D**) ascorbic acid (AsA) contents in leaves of two fragrant rice Guixiangzhan (GXZ) and Meixiangzhan-2 (MXZ-2) cultivars. Both fragrant rice cultivars were treated with two molybdenum levels: 0 µM (–Mo) and 1 µM (+Mo), against two cadmium levels: 0 µM (−Cd) and 100 µM (+Cd) in modified Hoagland solution. Vertical bar above indicates standard error of four replicates. Different lowercase letters (a, b, c, etc.) represent significant differences according to the LSD-test (*p* < 0.05, *n* = 4).

Interestingly, Mo supplementation mitigated the Cd toxicity effects under combined (Mo + Cd+) treatment and significantly increased the SOD (136.25% and 209.01%), POD (114.65% and 206.69%), CAT (148.56% and 129.68%), and APX (103.41% and 176.55%) activities in GXZ and MXZ-2 rice cultivars, respectively, as compared to Mo − Cd+ treatment (Figure 4). Moreover, significantly higher antioxidant enzymes activities in GXZ than MXZ-2 rice cultivar under concurrent (Mo + Cd+) treatment indicate that GXZ might efficiently utilize Mo more than MXZ-2 fragrant rice cultivar. Similarly, non-enzymatic antioxidant levels were also significantly suppressed under Cd stress as compared to sufficiently supplied (Mo+) treatments in both rice cultivars (Figure 5).

### 3.6. Effect of Mo and Cd on Antioxidant Gene Expressions

**Figure 6:**
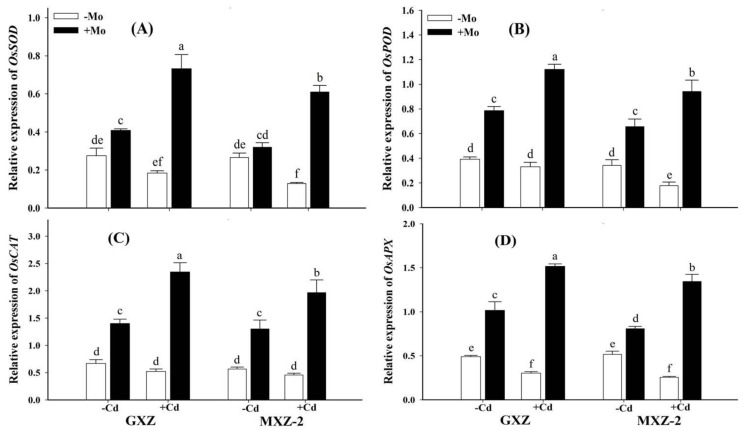
Influence of molybdenum (Mo) and cadmium (Cd) on qRT-PCR analysis of antioxidant enzyme-related transcripts of; (**A**) *superoxide dismutase (SOD)*, (**B**) *peroxidase (POD)*, (**C**) *catalase (CAT)*, and (**D**) *ascorbate peroxidase (APX)* in leaves of two fragrant rice Guixiangzhan (GXZ) and Meixiangzhan-2 (MXZ-2) cultivars. Both fragrant rice cultivars were treated with two molybdenum levels: 0 µM (–Mo) and 1 µM (+Mo), against two cadmium levels: 0 µM (−Cd) and 100 µM (+Cd) in modified Hoagland solution. Vertical bar above indicates standard error of four replicates. Different lowercase letters (a, b, c, etc.) represent significant differences according to the LSD-test (*p* < 0.05, *n* = 4).

Figure 6 illustrates the effects of Mo and Cd on antioxidant encoding genes expressions in two fragrant rice cultivars. Compared with +Mo, the expression of antioxidant encoding genes (*SOD*, *POD*, *CAT*, *APX*) in both rice cultivars was lower under Mo deprivation with or without Cd stress; however, the effects of Cd stress varied with Mo supply (Figure 6).

Under Cd toxicity, Mo supply enhanced the expression levels of *SOD, POD, CAT* and *APX* genes to 2.98, 2.39, 3.49 and 3.97 folds in GXZ and 3.73, 4.28, 3.30 and 4.25 folds in MXZ-2 rice cultivars, respectively, compared with Mo deprivation (Figure 6), suggesting that Mo supplementation performed a key role in alleviating Cd toxicity effects by strengthening the antioxidant defense system in fragrant rice cultivars. 

### 3.7. Correlation Analysis

**Figure 7:**
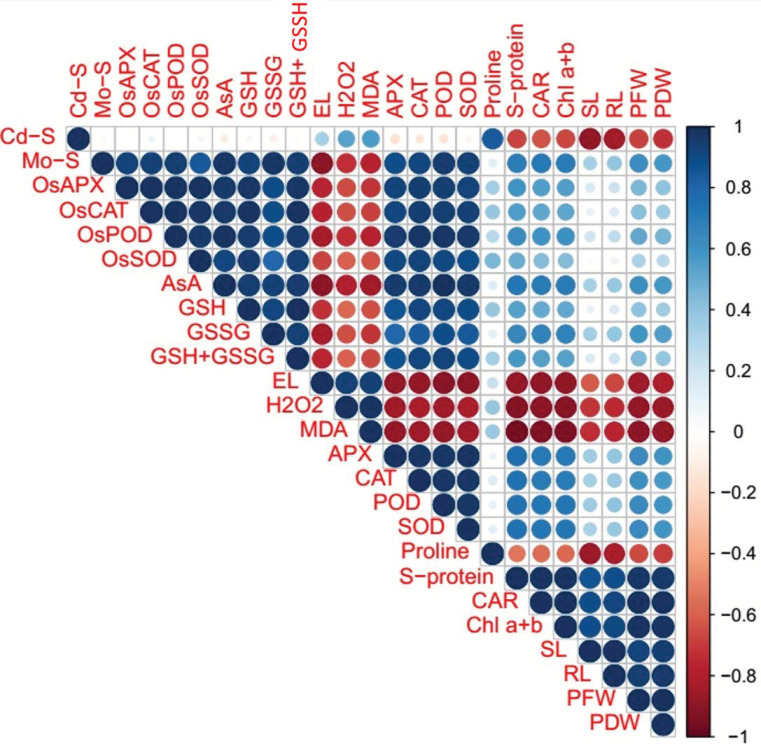
Correlation analysis between Mo and Cd application and growth attributes of fragrant rice cultivars. Cd-S (Cd concentration in shoots), EL (electrolyte leakage), H_2_O_2_ (hydrogen peroxide levels), POD (peroxidase activity), SOD (superoxide dismutase activity), APX (ascorbate peroxidase activity), CAT (catalase activity), MDA (malondialdehyde contents), SL (shoot length), RL (root length), PFW (plant fresh weight), PDW (plant dry weight), CAR (carotenoids content), Chl-a+b (total chlorophyll), S-protein (soluble protein), GSSG (oxidized glutathione), GSH (reduced glutathione), GSH+GSSG (total glutathione contents), Mo-S (molybdenum concentration in shoots) and AsA (ascorbic acid).

Pearson’s correlation analysis was performed to demonstrate existing relationships between combined application of Mo and Cd and different growth and biochemical attributes of fragrant rice cultivars (Figure 7). Cd concentration was positively correlated with the production of H_2_O_2_, MDA, EL and proline contents while it was negatively correlated with plant growth and biomass accumulation, photosynthetic pigments and S-protein contents; however, a weak correlation was observed between Cd concentration and antioxidant enzymes activities and transcript abundance. In contrast to Cd, Mo contents were positively correlated with plant height, biomass addition, photosynthetic pigments, S-protein, proline, enzymatic and non-enzymatic antioxidant responses of rice plants, while negatively correlated with H_2_O_2_, EL and MDA production. This correlation analysis demonstrated a close positive relationship between plant growth attributes and Mo concentrations while negative relations under Cd concentrations in fragrant rice cultivars.

## 4. Discussion

Heavy metals in general and Cd in particular often cause perturbations in various physiological, biochemical and molecular processes including stunted plant growth, photosynthesis, chlorophyll biosynthesis and antioxidant defense systems in plants [6,35]. Therefore, the alleviation of Cd toxicity in plant growth and developmental processes continues to be an important goal for plant scientists. Molybdenum (Mo), an essential and stress resistant microelement, has extended considerable attention due to its vital role in various plant growth and developmental processes and improving oxidative stress tolerance under drought, salinity, cold and heavy metal stresses [11,14,18]. The present study established an insight into the role of Mo application in modulating the morpho-physiological, biochemical and genetic responses of fragrant rice cultivars under Cd stress.

In the present study, Mo − Cd+ treatment severely hampered plant growth attributes in terms of plant height and biomass accumulations in both rice cultivars; however, the inhibition was more noticeable in MXZ-2 than GXZ cultivar (Table 2), and the reason might be GXZ cultivar is more resistant to Cd stress because it is well established that the production of plant biomass is an important indicator for evaluating plant tolerance to heavy metal stress [36]. However, Mo application significantly enhanced plant biomass in both fragrant rice cultivars and interestingly, GXZ recorded more pronounced effects under sufficient Mo supply than MXZ-2 cultivar, compared with –Mo treatments and these effects coincide with respective Mo concentrations (Table 3), suggesting that GXZ is more efficient in up-taking and utilizing Mo fertilizer than MXZ-2 fragrant rice cultivar. Moreover, in the present study, Mo supply alleviated Cd absorption (Table 3) and the possible reason is that uptake and transport of essential as well as heavy metals in plants are controlled by different metal transporters, for example, *IRT1* is responsible for uptaking iron, zinc manganese as well as toxic metal Cd from soil [37], while *HMA2* and *HMA4* pump Cd into the xylem and increase root-to-shoot translocation [38]. There are several reports in literature indicating that the increased expression level of *HMA2* or *HMA4* induced Cd xylem uploading for translocation to the shoots, leading to higher Cd content in plant shoots [39,40]. However, Mo application down-regulated *IRT1*, *HMA2* and *HMA4* genes expression and resulted in lowering Cd uptake in roots as well as Cd-translocation in stems, leaves and grains of *Brassica napus*, suggesting that Mo supply alleviates Cd absorption in plants [11].

A pronounced reduction in photosynthetic pigment contents was noticed in leaf tissues of Cd-stressed rice plants in the absence of Mo (Figure 1). These findings are concomitant with previous studies reporting severe decline of chlorophyll and carotenoids contents under Cd toxicity in *Oryza sativa* [24], *Phaseolus vulgaris* [41] and *Triticum aestivum* [2]. However, in the present study Mo supply to Cd stressed rice plants resumed Cd-induced loss in photosynthetic pigments. A possible explanation is that photosynthetic pigments are highly negatively correlated with MDA, EL and ROS in plant leaves and concomitantly in our results Cd concentration was positively correlated with ROS/MDA contents while negative relationships were observed for photosynthetic pigments, whereas Mo concentration showed contrasting relations (Figure 7). Moreover, Cd toxicity drastically inhibits the chlorophyll biosynthesis enzymes activities, such as protochlorophyllide reductase and δ-aminolevulinic acid dehydratase (ALA-dehydratase) [41] while Mo supply promotes ALA-dehydratase during the chlorophyll biosynthesis pathway [42]. Similarly, Mo-induced amelioration in photosynthetic pigments under stressed environments has also been reported previously under drought stress [32], low temperature stress [42] and ammonium stress [15].

In the present study, Cd stress decreased S-protein contents in rice cultivars (Figure 2A). The reason is that Cd toxicity increased the oxidative damage, as evident from higher H_2_O_2_, MDA and EL in leaf tissues (Figure 3), and it agrees with previous reports that Cd toxicity stimulates the S-protein degradation through increased protease activity [43] and excess ROS generation [44]. However, Mo supply significantly enhanced the S-protein contents in both fragrant rice cultivars, suggesting that Mo supplementation might have alleviated Cd-induced protein degradation possibly through prevention of ROS generation (Figure 3) and these results are concomitant with previous reports [16,32]. It is recognized that accumulation of proline is a noteworthy stress tolerance signal under heavy metals and also plays an important role in macromolecule stabilization, osmotic regulation, ROS-scavenging and cell fortification from oxidative harms [45]. Moreover, it regulates the enzymatic and metabolic activities and helps in stabilizing the protein integrity and is also considered a non-enzymatic antioxidant against stressful environments [46]. Therefore, the significant increase in proline contents in the combined (Mo + Cd+) treatment might be associated with maintenance of membrane integrity as compared to Cd toxicity (Figure 2B). 

In our experiment, Cd toxicity stimulated oxidative stress as evident from higher production of H_2_O_2_, MDA and loss of membrane integrity (greater EL) in both fragrant rice cultivars; nevertheless, harm in MXZ-2 was more prominent than GXZ rice cultivar (Figure 3). Cd toxicity often elicits the development of free radicals and ROS, causing ultra-structural and functional alterations in cell proteins, lipids, and DNA and cell nuclei. MDA, a primary sign of oxidative stress, is a by-product of polyunsaturated (decomposed) fatty acids while EL shows a loss in membrane fluidity. The reason is that Cd ions often interfere with bi-layers of lipids, induce leakage of K^+^ ions and lead to frequently modified or deformed cell membranes, thereby causing oxidative damages and increased MDA contents and EL. However, in this study Mo application decreased the contents of MDA, H_2_O_2_ and EL in leaves of both rice cultivars (Figure 3), which proves that Mo supplementation alleviates Cd-induced intracellular membrane damages in fragrant rice. These accommodative strategies of Mo to stabilize and sustain bilayer membranes and save cell membranes against oxidative stress damages agree with previous reports in strawberry [47] and wheat [48].

Plants depend on efficient enzymatic and non-enzymatic defense systems to quench superfluous ROS and maintain redox potential. The present study recorded differential responses of enzymatic, i.e., CAT, POD, SOD and APX, and non-enzymatic, i.e., GSH, GSSG and AsA, antioxidants in both rice cultivars and quite higher rates of activity were observed in GXZ cultivar than MXZ-2 (Figure 4 and Figure 5). The reason for greater antioxidant response in GXZ could be due to its effectiveness in dealing with Cd stress to a greater extent than MXZ-2 cultivar. SOD is an essential antioxidant enzyme specifically involved in O_2_^−^ to O_2_ detoxification and then to H_2_O_2_ [49], which is further reduced to H_2_O by CAT and POD. In addition, the shielding role of APX (a crucial antioxidant enzyme of ascorbate-glutathione cycle) helps in chloroplast cleanse H_2_O_2_ to H_2_O [50]. In this study, concurrent (Mo + Cd+) treatment triggered the activities of POD, SOD, CAT and APX (Figure 4), showing that Mo supplementation reinforced the enzymatic antioxidant defense system to safeguard rice plants against Cd-induced oxidative injuries. Similarly, non-enzymatic antioxidants such as GSH, GSSG and AsA also play key roles in scavenging ROS under stressful conditions and counteracting the oxidative stress of various heavy metals, which has already been mechanistically described in the Halliwell-Asada enzyme cycle. GSH shields guard cells from oxidative damages and has a significant role in reducing most ROS. However, when cells are exposed to increased levels of oxidative stress, GSSG will accumulate and the ratio of GSH to GSSG will decrease. Therefore, the determination of the GSH/GSSG ratio is a useful indicator of oxidative stress in cells and tissues [51]. In the present study, Mo application increased the GSH/GSSG ratio under Cd stress in both cultivars indicating that Mo supply alleviated Cd toxicity in fragrant rice seedlings. Taken together, it is inferred that Mo supply strengthened both enzymatic and non-enzymatic antioxidants to withstand against Cd toxicity in fragrant rice seedlings; however, reduced antioxidant activities under Cd stress might be due to superfluous ROS production, which may cause a reduction in their activities.

The heavy metals have brutally impaired the antioxidant defense system and more work focuses on quantitative analyses while gene expression is documented in few literatures [52,53,54]. To the best of our knowledge, no record exists on the transcript abundance of enzymatic antioxidant-related genes in fragrant rice under Mo supplementation against Cd toxicity. In this study Cd toxicity lowered the *SOD, POD, CAT* and *APX* genes expression levels (Figure 6), while combined (Mo + Cd+) application significantly up-regulated their transcript abundance, which also coincides with increased activities (Figure 4). Similarly, previous studies also reported that Mo application restricted the down-regulation of antioxidant-related gene expressions under drought [17] and ammonium stress [18]. Our findings, therefore, clearly suggest that Mo supplementation performed a key role in alleviating Cd toxicity through ROS-scavenging, modulating the antioxidant-defense system and related gene expressions in fragrant rice seedlings.

## 5. Conclusions

To the best of our knowledge, the present work offers the first demonstration of the physio-biochemical and molecular mechanisms concerning Cd-induced toxicity and Mo-mediated Cd-stress tolerance in fragrant rice cultivars. Cd-stress stimulated H_2_O_2_ production and electrolyte leakage, probably by desynchronizing the ROS scavenging system as supported by lower activities of SOD, POD, CAT and APX and gene expressions. However, Mo supplementation efficiently alleviated Cd-induced reductions in growth attributes of fragrant rice seedlings, which is primarily ascribed to decreased Cd uptake, fortification of photosynthetic pigments and proteins, efficient ROS-scavenging by strengthening both enzymatic and non-enzymatic antioxidant defense systems and related gene expressions. So, these findings explored that Mo application reinforced the antioxidant defense system and alleviated Cd-induced inhibitory effects on the growth performance of fragrant rice plants at the seedling stage. Nevertheless, future studies could be meditated under field conditions to examine Mo and Cd interactive effects on fragrance producing compounds and evaluate the grain quality responses in fragrant rice.

## Figures and Tables

**Table 1:** Sequences of primers used for qRT-PCR.

Genes	Strand	5’ to 3’ Primer Sequences	Annealing Temperature (Tm)	Accession no.
*OsSOD*	Forward	TGTCAACTGGACCACACTTC	58 °C	Os07g0665200
	Reverse	ACTTAAAACGCATGCACTCA		
*OsPOD*	Forward	CGACGATTTCTACGACTACAT	59 °C	Os10g0109600
	Reverse	TGATTGAGGAGGTTCTGGT		
*OsCAT*	Forward	GCACAGTTTGACAGGGAG	55 °C	Os06g51150
	Reverse	GTCTTTGGACTTGGCTTG		
*OsAPX*	Forward	TACGCCGACTTCTACCAGC	57 °C	Os07g0694700
	Reverse	TTTATTACAACCGCCACGA		
*ACTIN*	Forward	TGCCAAGGCTGAGTACGACGA	58 °C	Os03g50885
	Reverse	CAAGCAGGAGGACGGCGATA		

**Table 2:** Influence of molybdenum (Mo) supply on plant growth and biomass accumulation of fragrant rice seedlings exposed to different cadmium (Cd) levels.

Rice Cultivars	Treatments	Plant Growth (cm)		Plant Biomass (g plant^−1^)	
		Shoot Length	Root Length	Fresh Weight	Dry Weight
Guixiangzhan	Mo − Cd−	21.05 ± 1.09 ^c^	7.30 ± 0.73 ^c^	0.59 ± 0.026 ^c,d^	0.17 ± 0.014 ^c^
	Mo + Cd−	30.91 ± 1.78 ^a^	11.31 ± 0.68 ^a^	1.13 ± 0.084 ^a^	0.32 ± 0.027 ^a^
	Mo − Cd+	10.71 ± 0.69 ^d,e^	3.88 ± 0.35 ^e,f^	0.39 ± 0.038 ^e,f^	0.11 ± 0.006 ^d,e^
	Mo + Cd+	14.51 ± 1.36 ^d^	5.34 ± 0.61 ^d,e^	0.68 ± 0.060 ^c^	0.18 ± 0.011 ^c^
Meixiangzhan-2	Mo − Cd−	18.63 ± 1.55 ^c^	6.25 ± 0.32 ^c,d^	0.50 ± 0.033 ^d,e^	0.14 ± 0.011 ^c,d^
	Mo + Cd−	25.75 ± 2.14 ^b^	9.01 ± 0.53 ^b^	0.89 ± 0.106 ^b^	0.25 ± 0.021 ^b^
	Mo − Cd+	9.76 ± 0.75 ^e^	3.58 ± 0.31 ^f^	0.29 ± 0.024 ^f^	0.08 ± 0.005 ^e^
	Mo + Cd+	12.19 ± 0.84 ^d,e^	4.69 ± 0.35 ^e,f^	0.48 ± 0.018 ^d,e^	0.13 ± 0.008 ^d^

Note: (^a^) Mo − Cd− (0 µM Mo and 0 µM Cd), ^(b)^ Mo + Cd− (1 µM Mo and 0 µM Cd), ^(c)^ Mo − Cd+ (0 µM Mo and 100 µM Cd), (^d^) Mo + Cd+ (1 µM Mo and 100 µM Cd). Numerical values represent means ± S.E. from different independent treatments. Dissimilar superscripted letters (^a^, ^b^, ^c^, etc.) in each column indicate significant differences among different treatments at *p* < 0.05.

**Table 3:** Influence of molybdenum (Mo) supply on Mo and Cd concentrations in fragrant rice seedlings exposed to different cadmium (Cd) levels.

Rice Cultivars	Treatments	Mo Concentrations (µg g^−1^ DW)		Cd Concentrations (µg g^−1^ DW)	
		Root	Shoot	Root	Shoot
Guixiangzhan	Mo − Cd−	0.18 ± 0.01 ^c^	0.30 ± 0.04 ^c^	1.98 ± 0.26 ^d^	0.71 ± 0.05 ^d^
	Mo + Cd−	2.41 ± 0.17 ^a,b^	3.44 ± 0.11 ^a,b^	1.77 ± 0.13 ^d^	0.69 ± 0.07 ^d^
	Mo − Cd+	0.20 ± 0.02 ^c^	0.32 ± 0.02 ^c^	391.73 ± 21.98 ^a^	68.96 ± 7.10 ^a,b^
	Mo + Cd+	2.68 ± 0.24 ^a^	3.85 ± 0.43 ^a^	309.78 ± 26.62 ^b,c^	51.95 ± 2.74 ^c^
Meixiangzhan-2	Mo − Cd−	0.15 ± 0.01 ^c^	0.23 ± 0.03 ^c^	1.57 ± 0.18 ^d^	0.69 ± 0.04 ^d^
	Mo + Cd−	2.29 ± 0.13 ^b^	3.07 ± 0.07 ^b^	1.44 ± 0.11 ^d^	0.72 ± 0.05 ^d^
	Mo − Cd+	0.17 ± 0.01 ^c^	0.24 ± 0.02 ^c^	332.77 ± 27.54 ^b^	78.22 ± 4.91 ^a^
	Mo + Cd+	2.62 ± 0.17 ^a,b^	3.40 ± 0.22 ^a,b^	282.50 ± 15.34 ^c^	64.15 ± 4.98 ^b^

Note; ^(a)^ Mo − Cd− (0 µM Mo and 0 µM Cd), ^(b)^ Mo + Cd− (1 µM Mo and 0 µM Cd), ^(c)^ Mo − Cd+ (0 µM Mo and 100 µM Cd), ^(d)^ Mo + Cd+ (1 µM Mo and 100 µM Cd). Numerical values represent means ± S.E. from different independent treatments. Dissimilar superscripted letters (^a^, ^b^, ^c^, etc.) in each column indicate significant differences among different treatments at *p* < 0.05.
